# Levels of Integration in Cognitive Control and Sequence Processing in the Prefrontal Cortex

**DOI:** 10.1371/journal.pone.0043774

**Published:** 2012-08-29

**Authors:** Jörg Bahlmann, Franziska M. Korb, Caterina Gratton, Angela D. Friederici

**Affiliations:** 1 Helen Wills Neuroscience Institute, University of California, Berkeley, California, United States of America; 2 Max Planck Institute for Human Cognitive and Brain Sciences, Leipzig, Germany; 3 Center for Cognitive Neuroscience, Duke University, Durham, North Carolina, United States of America; Ecole Normale Supérieure, France

## Abstract

Cognitive control is necessary to flexibly act in changing environments. Sequence processing is needed in language comprehension to build the syntactic structure in sentences. Functional imaging studies suggest that sequence processing engages the left ventrolateral prefrontal cortex (PFC). In contrast, cognitive control processes additionally recruit bilateral rostral lateral PFC regions. The present study aimed to investigate these two types of processes in one experimental paradigm. Sequence processing was manipulated using two different sequencing rules varying in complexity. Cognitive control was varied with different cue-sets that determined the choice of a sequencing rule. Univariate analyses revealed distinct PFC regions for the two types of processing (i.e. sequence processing: left ventrolateral PFC and cognitive control processing: bilateral dorsolateral and rostral PFC). Moreover, in a common brain network (including left lateral PFC and intraparietal sulcus) no interaction between sequence and cognitive control processing was observed. In contrast, a multivariate pattern analysis revealed an interaction of sequence and cognitive control processing, such that voxels in left lateral PFC and parietal cortex showed different tuning functions for tasks involving different sequencing and cognitive control demands. These results suggest that the difference between the process of rule selection (i.e. cognitive control) and the process of rule-based sequencing (i.e. sequence processing) find their neuronal underpinnings in distinct activation patterns in lateral PFC. Moreover, the combination of rule selection and rule sequencing can shape the response of neurons in lateral PFC and parietal cortex.

## Introduction

The lateral prefrontal cortex (PFC) is linked to a variety of functions like cognitive control [1], working memory [2], rule learning [3], or language processing [4]. Cognitive control relates to the ability to work towards internal goals, while differentiating between conflicting thoughts or actions [5]. Abstraction is a crucial prerequisite of cognitive control [6]. The ability to process abstract action goals is necessary to flexibly act in changing environments. Abstraction in cognitive control can be investigated using task updating [7] or inhibition experiments [8], or working memory maintenance [9]. These tasks typically engaged mid lateral PFC regions, like inferior frontal sulcus (IFS), inferior frontal junction (IFJ), or middle frontal gyrus (MFG). Moreover, complex abstraction in cognitive control, like relational reasoning [10], sub-goal monitoring [11], or maintaining task-sets over time [12], [13] recruited rostral lateral PFC regions.

Sequence processing in language, on the other hand, refers to syntactic rules determining grammatical relations between elements. In language, the integration of single words into a phrase, and phrases into sentences are represented by sequence processing rules [14]. Convergent evidence suggest that ventrolateral PFC represents a core region for sequence processing of syntactic rules in natural languages [15]–[17] and in artificial grammars [18]–[20], for a recent review see [21].

So far, cognitive control and sequence processing has been investigated independently. However, both concepts have certain similarities. They have in common that lower levels of integration are combined into higher levels of integration. In language, lower level syntactic rules permit the integration of single words into phrases (e.g. phrase structure rules and local morpho-syntactic rules), and the integration of different phrases into sentences (higher-level syntactic rules). On the other hand, in cognitive control, lower levels of control engage the integration of contextual information in order to perform a given task (i.e. contextual control). This contextual information can be a cue (e.g. color or shape of an object) that determines the task to be executed. Higher levels of control necessitate the integration of task-set information over a certain amount of time (i.e. episodic control [13]). A typical higher-level control task is to maintain a cue-task association over a certain episode, which can vary across blocks [12].

Moreover, both concepts account for flexibility. In language, sequencing of syntactic rules leads to flexible combinations of words into sentences (“infinite use of finite means”; Humboldt, 1836, cited by [14]). In cognitive control, abstraction is assumed to facilitate flexibility in behavior to achieve goals in varying situations [1].

Most strikingly, experiments investigating into either of the two concepts have in common that they involve lateral PFC regions. However, sequential abstraction was shown to be restricted to ventrolateral PFC regions [18]–[20], [22] and cognitive control abstraction additionally recruited dorsolateral and rostral PFC [7], [12], [13], [23]–[26]. An interesting question is, whether cognitive control and sequence processing exist completely independent and in parallel, or whether they share certain similarities, not only conceptually like described above, but also neurally. The present study aimed to address this question. In particular, we investigated, whether cognitive control and sequence processing are represented in distinct or overlapping brain regions when investigated simultaneously.

We hypothesized that there is the possibility of distinct rostral and ventrolateral PFC regions engaged in the two processing types: complex sequential processing activates ventrolateral PFC [18], [20], [22] and complex cognitive control processing activates rostral PFC [7], [12], [13]. Alternatively, sequence and control processing might interact with each other in overlapping lateral PFC regions, such that complex sequential and complex control processing show different BOLD response characteristics in comparison to lower-order sequential and control processes.

**Figure 1:**
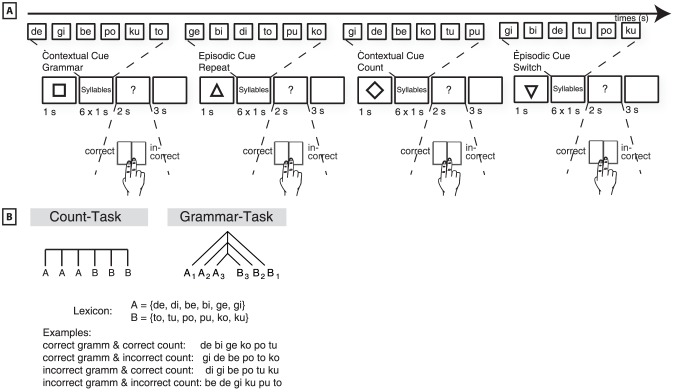
Description of experimental design. A: The square indicated to process the syllable sequences according to the artificial grammar (AG) rule (contextual-cue and grammar-task). Next, the upwards triangle indicated to repeat the AG task (episodic-cue and grammar-task). The diamond determined to apply the counting rule (contextual-cue and counting-task). Finally, the downwards triangle indicated to switch to the AG rule (episodic-cue and grammar-task). Syllables were presented each-by-each. B: Count-task: count /e/ syllables and match with /o/ syllables. Grammar-task: center-embedded processing of category A and category B syllables.

To test the two competing alternatives, we combined different levels of cognitive control processing with different levels of sequence processing. We applied a frequently used paradigm from the cognitive control research, namely the task-switching paradigm, together with a frequently used paradigm from sequence processing research, namely the artificial grammar (AG) task (see Figure 1). The lower level of cognitive control processing was manipulated using different cue-task associations (contextual-cue). The higher level of cognitive control processing was manipulated by using cues that determine whether to repeat the previous task or switch to the other task (episodic-cue). The lower level of sequence processing was implemented by grouping and matching classes of consonant-vowel syllables (count-task). Higher level sequence processing was necessary during the processing of a center-embedded organized AG rule (grammar-task). This experimental manipulation resulted in a 2×2 design with the factors CUE (contextual-cue versus episodic-cue) and the factor TASK (count-task versus grammar-task). We tested the two competing hypotheses in two steps. First, an ANOVA was applied on the BOLD response with the two factors CUE and TASK. Second, a multivariate approach of voxel-based tuning functions was applied on commonly activated brain regions.

## Materials and Methods

### Participants

This research was approved by the ethic committee of the Max Planck Institute for Human Cognitive and Neuroscience, Leipzig, Germany. Written informed consent was acquired prior to the scanning session for all participants. Twenty-one right-handed participants took part in the fMRI study (ten female, mean age = 25.2 years, SD = 2.1 years). They were all native speakers of German and had normal, or corrected to normal vision. None of the participants had a history of neurological, major medical, or psychiatric disorder.

### Materials

A 2×2 design was applied with the factors CUE and TASK. The factor CUE comprised of two types of explicit cues, namely a contextual-cue and an episodic-cue, triggering participant's choice between two tasks. The contextual-cue was directly linked to a particular task, i.e. grammar-task versus count-task. In contrast, the episodic-cue determined the choice of a task in dependency of the previous event, i.e. repeat the previously executed task or switch to the other task [27]. The factor TASK consisted of two different sequencing tasks. One task was to process a center-embedded AG rule (grammar-task), the other task was to count certain features of the stimulus sequence (count-task). Complex center-embedded structures can also be found in natural sentences (e.g. “The man the boy the dog bit greeted is my friend.”). In a recent study, natural sentences with three center-embeddings were compared with sentences of the same length comprising of long-distance dependencies, without center-embeddings [28]. In the present study, stimuli were consonant-vowel syllables that were presented each by each on the screen. Syllables were structured according to a center-embedded AG rule. This AG rule was first applied in a previous study [20]. Please see [20] for a detailed description of rule. In short, the sequencing rule was generated according to the formula A^n^B^n^, at which A and B were different types of syllable categories and n the number of items in a syllable sequence (e.g. n = 3: A_1_A_2_A_3_B_3_B_2_B_1_). Category A syllables ended with /e/ or /i/ and category B syllables ended with /o/ or /u/. Additionally, concatenations between the categories A_x_B_x_ were generated using plosives. The consonants /b/–/p/, /d/–/t/, and /g/–/k/ were grouped in order to generate a center-embedded structure (e.g. A_1_A_2_A_3_B_3_B_2_B_1_ = /be di ge ko tu pu/). Erroneous sequences comprised of six syllables, in which the concatenation between the categories was not matched (e.g. A_1_A_2_A_3_B_3_B_1_B_2_, see [20] for more details). All presented sequences had the same length of six syllables. The choice of the task was triggered by the cue immediately prior to the occurrence of the syllable sequence. The task was either to process the syllable sequence according to the artificial grammar rule (grammar-task), or to count the number of syllables ending with an /e/ and match this number with the number of syllables ending with an /o/ (count-task). For the grammar-task, participants were instructed to judge whether the syllable sequences followed the AG rule, or not. For the count-task, participants were instructed to judge whether the number of syllables ending with an /e/ was equal to the number of syllables ending with an /o/. Hence, both tasks could be responded to with a yes and a no option.

The cues were defined as follows: The contextual-cue comprised of either a square or a diamond. A square indicated to choose the grammar-task. A diamond indicated to choose the count-task. The episodic-cue comprised of either a triangle pointing upwards or pointing downwards. A triangle pointing upwards indicated to repeat the task that was previously accomplished (i.e. do the same task as before). A triangle pointing downwards indicated to switch from the previous task (i.e. do the other task as before).

Taken together in this 2×2 design the factors CUE (episodic-cue, contextual-cue) and TASK (grammar-task, count-task) were investigated. The combination of the two factors resulted in four experimental conditions, namely contextual-cue and grammar-task (CG), contextual-cue and count-task (CC), episodic-cue and grammar-task (EG), and episodic-cue and count-task (EC).

### Procedure

The tasks were learned two days prior to the fMRI session (please see [20] for a detailed description of the learning procedure). Participants first learned the two tasks separately. The grammar-task as well as the count-task was explained explicitly. In the next step, participants were introduced with the two cue types and performed the tasks again using the cues to choose between the two tasks. Learning ended when performance reached a criterion of 90% correct answered trials. The learning session took about 45 minutes. Immediately prior to fMRI session participants performed another training outside the scanner that lasted ca. 15 minutes. During the fMRI session participants were presented with 192 new syllable sequences. 96 items were linked with a contextual-cue, at half of which (48) squares were presented (indicating to choose the grammar-task) and the other half diamonds were presented (indicating to choose the count-task). The other 96 items were linked with an episodic-cue, half of which (48) with triangles pointing upward (repeat previous task) and the other half with triangles pointing downward (switch from previous task) was presented. Order of items was randomized, such that participants performed the grammar-task as often as the count-task, with the same number of grammatical and ungrammatical (grammar-task) and the same number of matching and miss-matching syllables (count-task), and the transitions between the tasks was counterbalanced. Each cue was presented for 1000 ms in the middle of the screen, followed by 6 syllables, presented 1000 ms, one after another. After the presentation of a sequence, participants were asked to provide a judgment regarding the grammaticality of the sequence within 2000 ms, followed by feedback for 500 ms (i.e. the word “correct” or “wrong” was presented in green or red on the screen). Afterwards a fixation cross was shown for a further 3000 ms (see Figure 1). Trials started with a jitter of 0, 500, 1000, or 1500 ms. Additionally, 48 null-events (presentation of a fixation cross, same jittering and length as trials) were randomly interspersed.

### fMRI Image Acquisition

Imaging was performed on a 3T scanner (Medspec 30/100, Bruker, Ettlingen). Stabilization cushions were used to reduce head motion. For registration purposes, two sets of two-dimensional anatomical images were acquired for each participant immediately prior to the functional imaging session. An MDEFT (data matrix 256×256, TR = 1.3 s, TE = 7.4 ms) and an EPI-T1 (TE 14 ms, TR 3000 ms) sequence were used. Additionally, geometric distortions were characterized by a B0 field-map scan. The field-map scan consisted of a gradient-echo readout (32 echoes, inter-echo time 0.64 ms) with a standard 2D phase encoding. The B0 field was obtained by a linear fit to the unwrapped phases of all odd echoes. Functional MRI scanning was carried out using a T2^*^-weighted BOLD sensitive gradient echo echo-planar imaging sequence (TR = 2 s, TE = 30 ms, FOV = 19.2 cm, 64×64 matrix, resulting in an in-plane resolution of 3 mm×3 mm). Thirty slices (thickness: 3 mm with an interslice gap of 1 mm) covering the whole brain were acquired. Anatomical and functional images were positioned parallel to AC-PC. One functional run with 1442 volumes was collected. The fMRI session lasted ca. 50 minutes.

### Image Processing

MRI data were analyzed using SPM8 (available at http://www.fil.ion.ucl.ac.uk/spm) on a PC workstation. Pre-processing comprised realignment and unwarp, slice timing, coregistration, segmentation, normalization to MNI space, and smoothing with a 8 mm full-width at half-maximum (FWHM) Gaussian kernel. Estimation of geometric distortion parameters for the realignment and unwarp procedure was conducted using the individual field maps. Normalizing an individual structural image to the SPM8 T1 brain template was processed in two steps: First, estimation of the normalization parameters, and second, writing the normalized images with the parameters. This parameter transformed the structural images and all EPI volumes into a common stereotactic space (i.e. MNI stereotactic space) to allow for across-subject analyses. Voxel size was interpolated during pre-processing to isotropic 3×3×3 mm.

### Univariate Statistical Analysis

For the statistical analysis, the onset from each condition was modeled as an event of interest with duration of zero. Additionally, also error trials (i.e. trials in which participants responded with the incorrect button press or not at all) and the baseline (fixation cross) were modeled as distinct conditions and were not mixed with the four conditions of interest. The six movement parameters were included as regressors in the design matrix. Confounds by global signal changes were removed by applying a high pass filter with a cut-off frequency of 128 seconds. In total, there were up to 48 events per condition. In the context of the general linear model, these events were convolved with a synthetic hemodynamic response function, yielding statistical parametric maps [29]. The onset of each event was set to the cue presented before each syllable sequence. Signal change relative to baseline in each condition was estimated using statistical parametric maps of the T-statistics. The resulting individual contrast images of each condition were submitted to the second level analysis. First, a 2×2 ANOVA with the factors CUE (contextual-cue versus episodic-cue) and TASK (grammar-task versus count-task) as implemented in SPM8 was applied. Additionally, a conjunction analysis between the factors CUE and TASK was conducted: Activation pattern resulting from the two main effects were conjoint using the ‘conjunction null’ method (equivalent to logical AND) as implemented in SPM8 [30]. To protect against false-positive activations a double threshold was applied, by which only regions with a z-score exceeding 3.09 (p<0.001, uncorrected) and a minimum cluster size threshold of 34 adjacent voxels were considered (corresponding to p<0.05, corrected). This was determined in a Monte Carlo simulation using the tool AlphaSim included in AFNI (http://afni.nimh.nih.gov/pub/dist/doc/manual/AlphaSim.pdf). This procedure represents an alternative to the Bonferroni-correction for multiple comparisons. This program calculates the number of voxel cluster and approximates the number of adjacent voxel per cluster necessary for multiple comparison correction.

The timecourse analysis was conducted with the Marsbar toolbox (http://marsbar.sourceforge.net/). Data for timecourse analysis was extracted from a 6-mm radius spherical volume. In each participant's data the centers of the regions of interest (ROIs) were set to the local maximum of the peak voxel in the brain areas that were identified in the interaction between factors TASK and CUE.

### Multivariate Statistical Analysis

Multivariate methods such as the construction of voxel tuning functions have been recently applied in visual perception studies, in order to disambiguate effects that occur at a voxel level, even if they are below threshold in a standard statistical analysis [31]. Conventional fMRI methods are not capable of detecting these fine-tuned changes, because the quite large voxel resolution (mostly 3×3×3 mm) is not sensitive enough to detect submillimeter columns of neurons that are selective for different visual features (e.g. orientations, colors, or objects). However, recent studies suggest that even if each voxel contains neurons tuned to many different visual features, the overall activation level is mostly predicted by the dominant tuning preference of the neurons within that voxel [32], [33].

Here we apply this voxel tuning analysis to the study of tuning in complex cognitive processes, in order to detect fine-tuned neural modulations in lateral frontal and parietal cortex. The univariate analysis revealed no significant differences in BOLD response in these areas between sequence processing and cognitive control processing. A similar timecourse difference between grammar versus count processing (TASK) and episodic versus contextual (CUE), would suggest no interaction between these two types of processes. Voxel tuning analysis may have more sensitivity to distinguish how voxels are tuned to these different conditions. Thus, in the present study a multivariate pattern analysis and a computation of tuning functions was applied. This method was first described to identify selectivity of voxels within sub-regions in the human visual cortex to different orientations [31]. Here, we used this method for the first time to detect differences in complex cognitive functions in frontal brain regions. Pattern analysis and voxel tuning functions were conducted using Python (http://python.org) and NumPy (http://numpy.scipy.org).

#### ROI selection

Functional ROIs were defined based on the F-Test (effect of interest) of the ANOVA analysis described above, in order to identify voxels that responded maximally to all experimental conditions. This procedure ensured that ROI selection was orthogonal to the consequent pattern analysis [34]. Activation cluster were identified with the threshold of p<0.05 (family-wise error correction, FWE) and 50 consecutive voxels (see Table 1 for the number of voxels in each ROI). ROIs were extracted using Marsbar toolbox (http://marsbar.sourceforge.net/).

**Table 1:** Whole brain ANOVA.

Brain region	BA	Size	x	y	z	Z _max_
L lateral frontal cortex	6/44/45	585	−42	26	19	7.24
L parietal lobe	7/40	629*	−27	−61	40	7.32
L ITG	37	130	−51	−55	−17	6.86
L IOG	18	159	−21	−94	−8	6.64
R Cerebellum		306	27	−67	−26	5.29

Effect of Interest (F-Test).

Anatomical areas, approximate Brodmann's Area (BA), number of activated voxel in cluster (Size), mean x, y, and z Montreal Neurological Institute (MNI) coordinates, and maximal *Z* values of the significant activations are presented.

L, left hemisphere; R, right hemisphere; ITG, inferior temporal gyrus; IOF, inferior occipital gyrus.

* p<.001 (FWE).

#### Multivariate pattern analysis

For the multivariate pattern analysis the experiment was divided into six arbitrary runs for cross-validation of the tuning curve classes. We investigated four experimental conditions: contextual-cue and grammar-task (CG), contextual-cue and count-task (CC), episodic-cue and grammar-task (EG), and episodic-cue and count-task (EC). Each experimental condition consisted of 48 trials. Eight trials from the 48 trials per condition were randomly assigned to one run. Thus, a single trail was assigned to only one run (no overlap of trials between analysis runs). This was done for all four conditions (i.e. CG, CC, EG, and EC). The random assignment of trials to runs was different in all 21 subjects. Erroneous trials were randomly assigned to the six different runs (in cases in which participants committed errors). In each run, trials not assigned to this run (including erroneous trials, baseline trials, and the 40 non-assigned trials) were treated as one regressor of no interest. The six movement parameters were also included as regressors. A general linear model on unsmoothed data was applied as implemented in SPM8, yielding statistical parametric maps. In each ROI, beta-values for each voxel were extracted. Raw voxel values were normalized using a z-transformation, to remove differences in mean signal intensity.

#### Voxel tuning functions

In each ROI, beta-values from all but one run were extracted to form a classification set. In each classification set, beta-values of the four experimental conditions were compared. The preference of each voxel was determined based on the condition that evoked the largest mean response (highest beta value) across classification runs. This was done independently for each voxel, without fixing the number of voxels that could be tuned for each condition. The final run (the analysis run; not used for classification) was then used to extract the responses of this voxel to all four conditions and construct a voxel tuning curve. A selection step was introduced at this stage that selected only voxels showing reliable classification for analysis: in order to be reliable, a voxel had to show the same condition preference in at least half of the classification runs. This procedure was repeated using a ‘leave-one-run-out’ cross-validation for all combinations of classification and analysis runs. Data from each ROI was analyzed separately. The resulting voxel tuning functions in each ROI represent a measure of the preference of a region to a particular experimental condition.

## Results

### Behavioral Results

**Figure 2:**
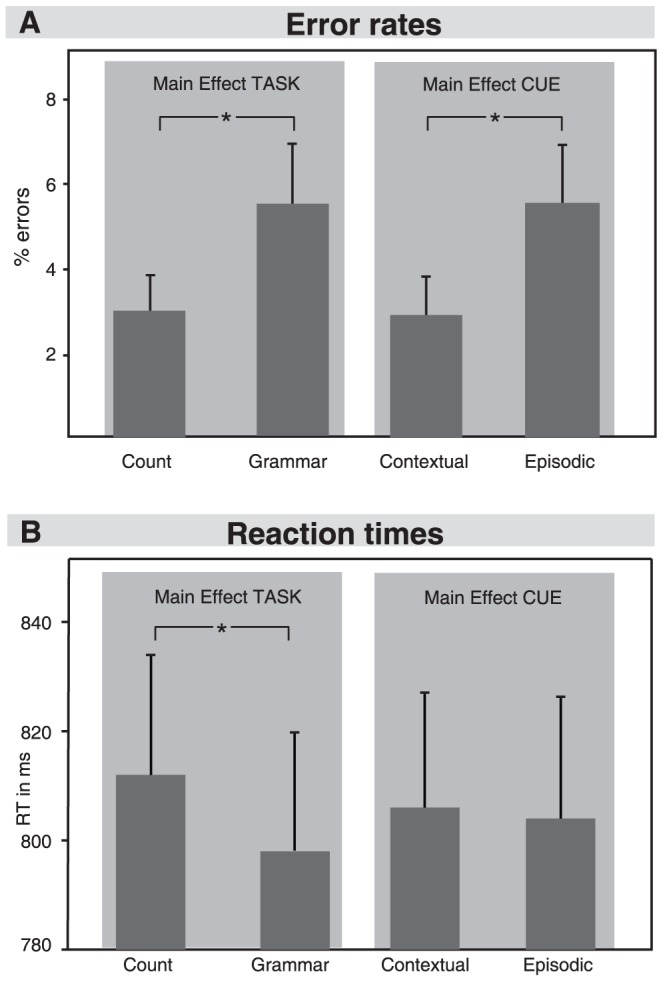
Behavioral results. A: Error rates of the two TASK conditions (count-task and grammar-task) and the two CUE conditions (contextual-cue and episodic-cue). B: Reaction times of the same conditions.

Mean accuracy was near ceiling for all four conditions: 4.25% (SD = 5.45) of all stimuli were judged erroneously (see Figure 2). An ANOVA with factors TASK (grammar-task versus count-task) and CUE (contextual-cue versus episodic-cue) was conducted on error rates. This analysis revealed a main effect of CUE (F(1,20) = 7.53, p<.05) and a main effect of TASK (F(1,20) = 5.62, p<.05). The step down analysis showed that participants committed more errors in grammar-task (5.5%) than in count-task (3.0%). Additionally, more errors were committed after an episodic-cue (5.6%) than after a contextual-cue (2.9%). The interaction was not significant. An ANOVA on reaction times with the same factors revealed a main effect of TASK (F(1,20) = 4.46, p<.05), indicating that participants reacted faster in grammar-task (798 ms) than in count-task (812 ms). In order to investigate whether a speed accuracy tradeoff could explain faster reaction times and increased error rates during grammar-task in comparison to count-task, further analyses were conducted. We correlated individual reaction times with corresponding error rates for all grammar-task trials and for all count-task trials separately. A significant negative correlation between individual reaction times and error rates would predict that participants who conduct many errors, would also be fast in their response (and vice versa). This was not the case; correlation coefficients were very low (r_grammar-task_ = −0.213, r_count-task_ = −0.048). Moreover, error rates were very small (e.g. two participants did not conduct any errors) so that the data sample was not heterogeneously distributed and a ceiling effect could explain the significant results.

### fMRI Results

**Figure 3:**
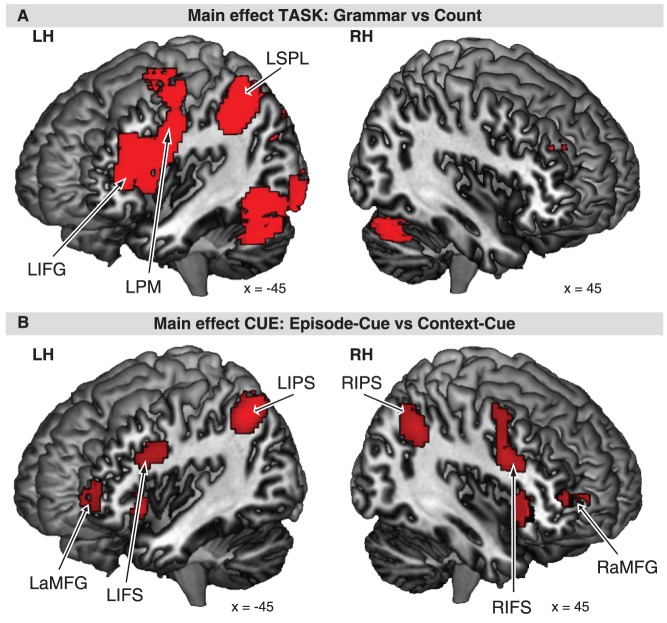
fMRI results. A: Main effect of TASK revealed increased hemodynamic responses in left inferior frontal gyrus (LIFG), left premotor cortex (LPM), left superior parietal lobe (LSPL), and right Cerebellum. B: Main effect of CUE engaged bilateral anterior middle frontal gyrus (LaMFG, RaMFG), inferior frontal sulcus (LIFS, RIFS), and inferior parietal sulcus (LIPS, RIPS).

**Figure 4:**
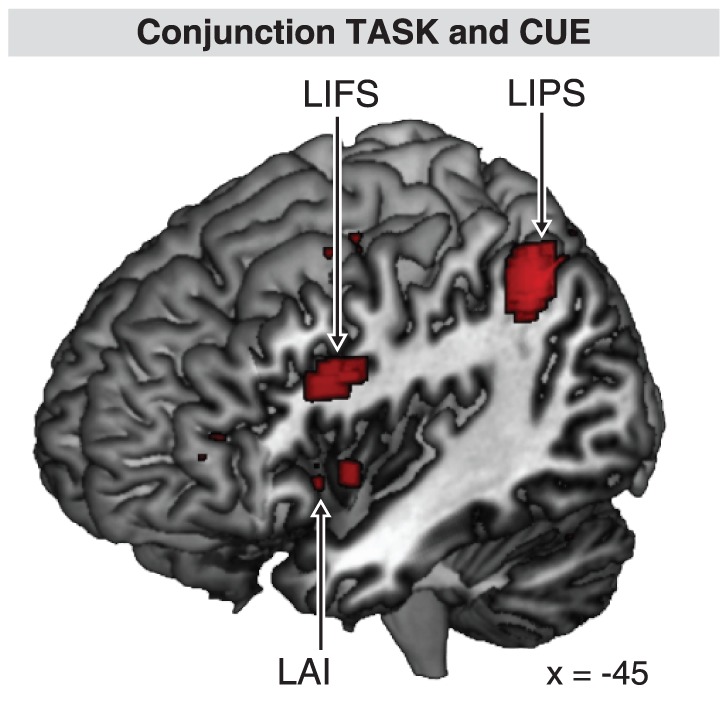
Conjunction analysis of the main effect TASK and main effect CUE. A conjoint activity in the left hemispheric inferior parietal sulcus (LIPS), inferior frontal sulcus (LIFS), and anterior insula (LAI) was observed.

Main effect of TASK exhibited a mostly left hemispheric activation pattern (see Table 2, Figure 3), including inferior frontal gyrus (IFG, pars opercularis), premotor cortex (PM), IFS, superior parietal lobule (SPL), middle occipital gyrus, precuneus, SMA, Thalamus, and Pallidum. In the right hemisphere, Cerebellum, Putamen, and Pallidum were activated. The cortical network revealed by main effect of CUE engaged bilateral anterior middle frontal gyri (aMFG), bilateral IFS, bilateral anterior insular cortices (AI), bilateral inferior parietal sulci (IPS, and left cerebellum (see Table 2, Figure 3). The interaction of TASK and CUE caused activation in several areas, including right mid-occipital gyrus, bilateral precentral gyri, bilateral Thalamus, right cerebellum, left medial superior frontal gyrus, and white matter (see Table 2). The conjunction analysis of factors TASK and CUE revealed common activity in bilateral AI, left IFS, and left IPS (see Table 2, Figure 4).

**Table 2:** Whole brain ANOVAs.

Brain region	BA	x	y	z	Z _max_
**Main effect TASK**					
L IFG opercularis	44	−54	11	22	6.53
L IFS	48/45	−42	26	19	7.63
L Premotor C	6	−39	−1	58	7.18
L SPL	7/40	−27	−61	43	7.79
L SMG	48	−60	−22	22	4.36
L SMA	6	−6	5	55	4.98
R MFG	46	33	44	34	5.18
L Thalamus		−12	−16	7	6.24
R Putamen		21	−22	−11	4.69
L Pallidum		−18	−4	−2	5.14
R Pallidum		18	−10	−2	5.55
R Cerebellum		27	−67	−26	6.98
**Main Effect CUE**					
L IFS	44/48	−45	17	25	3.70
R IFS	44/46	51	23	34	4.36
L IPL	40	−45	−52	46	4.96
R IPS	40	48	−43	37	4.04
L aMFG	10/46	−39	53	1	3.50
R aMFG	10/46	39	53	7	3.49
L anterior Insula	48	−30	23	−5	4.83
R anterior Insula	48	30	23	−5	4.55
L Cerebellum		−12	−79	−29	4.53
**Interaction CUE×TASK**					
R mid Insula/white matter	48	27	−22	1	4.74
R mid occipital G	18	30	−88	13	4.19
R Caudate/white matter		24	2	25	4.17
L precentral G	4	−18	−28	73	5.01
L/R Thalamus		0	−10	10	3.64
R Cerebellum (6)	18	9	−64	−14	3.58
R mid Cingulum/white matter		6	−4	31	3.50
**Conjunction CUE ∩ TASK**					
L IPS	40	−42	−52	46	4.84
L anterior Insula	47	−30	23	−2	4.55
R anterior Insula	47	30	23	1	3.69
L IFS	44/48	−45	17	25	3.70

Anatomical areas, approximate Brodmann's Area (BA), mean x, y, and z Montreal Neurological Institute (MNI) coordinates, and maximal *Z* values of the significant activations are presented.

L, left hemisphere; R, right hemisphere; IFG, inferior frontal gyrus; SPL, superior parietal lobe; MFG, middle frontal gyrus; SMG, supra-marginal gyrus; IPS, inferior parietal sulcus; aMFG, anterior middle frontal gyrus.

### Time course analysis

The activation pattern of the interaction effect was not predicted a-priori. In order to further evaluate these effects, we explored the hemodynamic responses in those regions that showed a significant interaction effect in the whole-brain analysis (see Table 2). Visual inspections revealed that all regions showed a deactivation of hemodynamic response relative to baseline.

### Voxel tuning analysis

**Figure 5:**
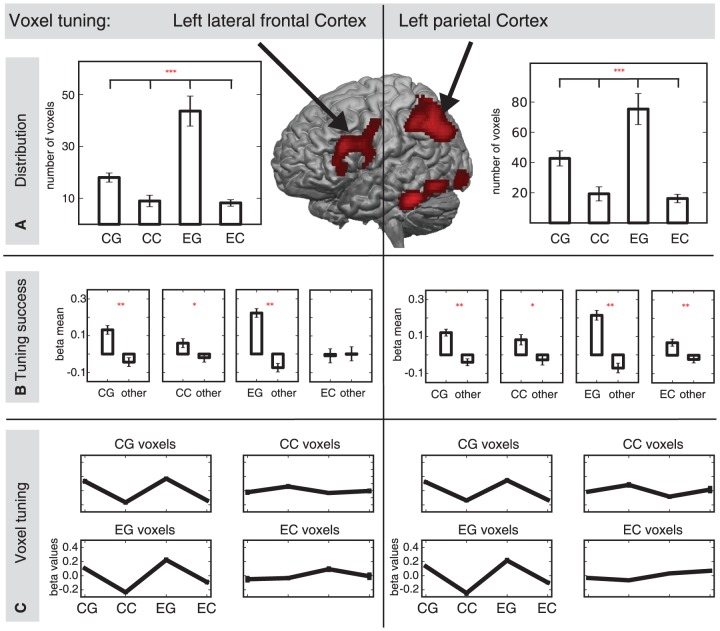
Voxel tuning in Left lateral frontal and Left parietal Cortex, separately for the four conditions. A: Distribution of number of voxels that were found to be tuned for one of the four conditions. Historgrams show the proportion of voxels that respond maximally to each condition. B: Tuning success measured by the comparison of mean beta values of voxels that were tuned for one condition against mean beta values of voxels of the other three conditions. C: Voxel tuning of each condition separately compared to the other three conditions. CG = contextual-cue and grammar-task, CC = contextual-cue and count-task, EG = episodic-cue and grammar-task, EC = episodic-cue and count-task. * … p<.05; ** … p<.01; *** … p<.001.

First, we investigated the number of voxels specifically tuned to one of the four conditions (i.e. contextual-cue and grammar-task (CG), contextual-cue and count-task (CC) episodic-cue and grammar-task (EG), and episodic-cue and count-task (EC)). In order to explore the overall preference of a region to a condition, an ANOVA with the factors CUE and TASK on the number of tuned voxels was conducted in each ROI (see Figure 5A). An interaction between CUE and TASK was found in left lateral frontal cortex (F(1,20) = 27.09, p<.001], in left parietal lobe [F(1,20) = 40.5, p<.001], and in right Cerebellum [F(1,20) = 18.81, p<.001]. Paired sample t-Tests on the four conditions were conducted based on this interaction. The four experimental conditions were contextual-cue and grammar-task (CG), contextual-cue and count-task (CC) episodic-cue and grammar-task (EG), and episodic-cue and count-task (EC). This analysis revealed that significantly more voxels were tuned for the EG condition than for the CG condition, in left lateral frontal cortex [t(20) = 6.24, p<.001], in left parietal lobe [t(20) = 2.98, p<.05], and in right Cerebellum [t(20) = 4.51, p<.001]. Also significantly more voxels were tuned for the CG condition than for EC and CC conditions, in left lateral frontal cortex [EG vs EC: t(20) = 4.03, p<.001 and EG vs CC: t(20) = 4.39, p<.001], in left parietal lobe [EG vs EC: t(20) = 4.09, p<.001 and EG vs CC: t(20) = 4.57, p<.001], and in right Cerebellum [EG vs EC: t(20) = 4.52, p<.001 and EG vs CC: t(20) = 4.04, p<.001]. These regions also showed a main effect of CUE and a main effect of TASK. A main effect of CUE and a main effect of TASK was found in left lateral frontal cortex (CUE [F(1,20) = 11.38, p<.001] and TASK [F(1,20) = 24.04, p<.001]), in left parietal lobe (CUE [F91,20) = 14.02, p<.001] and TASK [F(1,20) = 33.89, p<.001]), and right Cerebellum (CUE [F(1,20) = 21.6, p<.001] and TASK [F(1,20) = 34.46, p<.001]). Additionally, a main effect of TASK was also observed in left inferior temporal cortex [F(1,20) = 13.42].

Second, the overall tuning success of the four different experimental conditions was investigated (see Figure 5B). In this analysis we explored how well the classifier does in detecting voxels in a ROI that are specifically tuned for one condition. To do so, a paired sample t-Test of the condition of interest against the mean of the other three conditions was conducted on the beta values in each voxel. A significant difference between beta values of the condition of interest against all other conditions would speak for the selectivity of these voxels for a condition. This analysis revealed a significant difference between the single conditions against the other conditions in most of the ROIs [t-values between 1.7 and 6.8]. Tuning success was not significant in the EC condition in left lateral frontal cortex, right Cerebellum, and left inferior temporal cortex [t-values<1, ns]. Note that beta values plotted in Figure 5B have been normalized to the mean response of each voxel, which is why they may overall appear to be around 0.

## Discussion

The present study aimed to investigate cognitive control with sequence processing in an integrative paradigm. One hypothesis was that distinct lateral PFC regions are engaged in the two types of processing, such that ventrolateral PFC is activated during sequence processing [18], [20], [22] and rostral PFC regions are recruited during cognitive control [7], [12], [13]. Alternatively, both types of processing may interact in overlapping rostral and ventrolateral PFC regions. To test the two alternative hypotheses we used two different task cue sets to manipulate cognitive control, and two sequencing rules that differed in complexity to vary sequence processing. Consistent with previous studies, we found that increases in complexity of sequencing rules were associated with activation in left ventrolateral PFC (IFG) and increased cognitive control processing engaged bilateral dorsolateral and rostral PFC regions. Activation pattern of cognitive control and sequence processing partially overlapped in left lateral PFC, however, they did not interact with each other in this region. However, an interaction between cognitive control and sequence processing was found in left lateral frontal and parietal areas when applying a multivariate voxel tuning analysis.

The multivariate voxel tuning analysis revealed that most voxels in left lateral frontal and parietal cortex were tuned for the processing of the more complex cognitive control task (episodic-cue) and the more complex sequencing rule (grammar-task, see Figure 5A). A smaller number of voxels in these areas was tuned for the processing of the less complex cognitive control task (contextual-cue) and the more complex sequencing rule (grammar-task). The smallest number of voxels was tuned for the processing of the contextual-cue and grammar-task as well as the contextual-cue and count-task. This interaction between cognitive control and sequence processing indicates that left lateral frontal and parietal areas are engaged during both processing types. In addition, both processing types influence each other in an additive way. The data at hand suggests that voxel tuning for complex cognitive control and complex sequence tasks in lateral frontal and parietal cortex is evidence for the adaptive nature of neurons in these regions. Neurons flexibly adapt to changing requirements during cognitive control of rule selection as well as during sequence processing according to a given rule. This flexibility is reflected by the number of voxels tuned for the combined, complex rule selection and complex rule processing. These results accord well with the suggestion of an adaptive coding network comprising of the PFC and parietal cortex [35], such that activation in this network adaptively changed under different task-demand conditions in the present study (see also [36] for recent empirical work on adaptive coding). Furthermore, individual voxels tend to show similar tuning for the grammar-task, regardless of cognitive control condition (see Figure 5C). These results might suggest that sequence processing recruits left lateral frontal and parietal areas stronger than cognitive control processing.

Complex sequence processing was implemented using the AG rule A^n^B^n^ that generated center-embedded sequences (e.g. n = 2; A_1_A_2_B_2_B_1_). This rule can simulate natural center-embedded sentences like [John(A_1_), who(A_2_) was tall(B_2_), liked Mary(B_1_).]. We used an AG rule in order to maximally control for confounding factors like unsystematic semantic and phonological variability of experimental stimuli. In recent years a growing amount of studies were published dealing with the learning and processing of AG rules [18], [19], [37]–[41]. In the present study, the processing of a center-embedded AG rule in comparison to a counting condition correlated with activity in left ventrolateral PFC (IFG). This effect was independent of the cue type (episodic-cue or contextual-cue). These results are consistent with our previous findings, suggesting a crucial role of the left ventrolateral PFC during the processing of complex artificial grammars, which was shown for language related stimuli (i.e. sequences of consonant-vowel syllables, [20]) as well as for visuo-spatial stimuli (i.e. sequences of non-world objects with different shapes and surface structures, [42]). Our results are also compatible with the findings of a study by Tettamanti and colleagues [41] who found the left ventrolateral PFC to be sensitive for processing of long-distance dependencies between constituents in sentences, but also between elements of non-linguistic symbols. Taken together, these results suggest a domain-general involvement of the left ventrolateral PFC during complex sequence processing.

Some researchers had claimed that the center-embedded AG rule at hand could also be mastered by using simpler processing strategies like counting and matching of syllables [37], [40]. Under this view, it could be argued that the activity in left ventrolateral PFC might reflect phonological processes due to the counting of syllables (or other alternative strategies) and not complex structure processing. However, note that characteristic of the present task was that during the counting condition participants count the vowels /e/ and match it with the vowels /o/. By directly comparing the processing of a center-embedded AG rule condition with the counting condition we clearly show the engagement of the left ventrolateral PFC. This allows the conclusion that the left ventrolateral PFC is primarily involved in the processing of center-embedded AG rules, or at least more so than in more simple phonological processes like counting.

In the present study, complex cognitive control was manipulated through the usage of cues that determined whether to switch from the previous task or repeat the previous task (episodic-cue). Recent fMRI experiments that focused on complex cognitive control processes investigated task-set preparation [43], relational integration [10], relational reasoning [44], maintaining or switching of task-sets over time [13], embedding of stimulus-response chunks [22], or a variation of control demands necessary for selection of task-sets [12]. Despite of the differences in theoretical motivations and types of experimental manipulations, these studies have in common that increased cognitive control processing engage rostral areas of the lateral PFC. Our results strongly converge with these findings: Increased cognitive control processing, as reflected by the episodic-cue, indicated activation of rostral PFC regions (bilateral anterior middle frontal gyri, BA 10/46). Thus, our findings substantiate results from previous studies [10], [12], [13], [43], [44], suggesting a strong engagement of rostral PFC regions during complex cognitive control processes.

Another important finding of the present experiment was that activations of cognitive control emerged bilaterally and activations of sequence processing engaged mostly left hemispheric brain regions. Also, complex control processing engaged rostral and ventrolateral PFC regions whereas complex sequence processing was restricted to ventrolateral PFC (see Figure 3). Previous findings demonstrate that cognitive control experiments (i.e. task switching experiments) using numerical tasks engage the superior parietal sulcus [27] and motion discrimination tasks engage area MT [45], in addition to lateral PFC activations. Here we demonstrate that a cognitive control experiment using a sequencing task recruits the ventrolateral PFC in addition to rostral PFC. These findings suggest a functional segregation of the cognitive control and sequence processing, because they recruit dissociable prefrontal regions. However, we also showed that a conjoint left hemispheric network comprising ventrolateral PFC as well as inferior parietal regions was recruited during complex sequential and complex control processes (see Figure 5). These results suggest that even if a common neuronal network was recruited during sequential and control abstraction, they appear to be functionally discrete. Cognitive control and sequence processing seem to be processed in parallel and independently in the lateral PFC.

In a recent study, Koechlin and Jubault [22] proposed a hierarchical organization of the ventrolateral PFC. In this experiment, participants performed motor responses to letter sequences that comprises of simple chunks or superordinate chunks. As a results a caudal-rostral gradient for simple – superordinate chunks was found in bilateral PM and IFG. The present results are only partly compatible with the study by Koechlin and Jubault (2006), since we did not find such a gradient for sequential abstraction, though both studies used language-related stimuli (i.e. letters and syllables). A fundamental difference between the two studies is, however, that Koechlin and Jubault (2006) systematically varied cognitive control, but not sequence processing. In contrast, the study at hand systematically varied both, cognitive control and sequence processing. Thus, even if a caudal – rostral gradient was detected for cognitive control using language-related stimuli (Koechlin and Jubault, 2006), this gradient remains to be demonstrated for different levels of sequence processing.

Taken together, on the one hand, our findings speak in favor for a multi-functional architecture of the PFC, such that similar PFC regions are recruited during different task requirements. Processing of letter sequences and processing of task cues engaged overlapping left frontal brain regions as indicated by the conjunction analysis. Moreover, the majority of voxels in lateral frontal and parietal brain regions were recruited during processing of the combination of complex rule selection and complex sequence processing, suggesting that the two different processes are integrated in these regions. On the other hand, the dissociable pattern of frontal activity for rule selection (cognitive control) in the dorso-lateral and rostral PFC and rule processing (sequence processing) in the ventro-lateral PFC suggest that the two types of processing recruit different neural networks. Overall, our results demonstrated the adaptive nature of some lateral PFC regions: the area can be engaged in two different processes (as indicated by conjoint activation and interaction in voxel tuning), but in addition distinct brain regions are recruited during the two processes (i.e. dissociated activation pattern for cognitive control and sequence processing).
